# Modulation of *TLR7*, *TYK2* and *OAS1* expression during SARS-CoV-2 infection

**DOI:** 10.3389/fimmu.2025.1713928

**Published:** 2025-12-09

**Authors:** Estíbaliz Alegría-Carrasco, Marta Jaén-Castaño, Pablo Delgado-Wicke, Nelly D. Zurita-Cruz, Nuria Montes, Emilia Roy-Vallejo, Sara Fernández de Córdoba-Oñate, Ana Nicolao-Gómez, Rosa Carracedo-Rodríguez, Ana Marcos-Jiménez, Laura Cardeñoso-Domingo, Isidoro González-Álvaro, Elena Fernández-Ruiz

**Affiliations:** 1 Molecular Biology Unit, La Princesa University Hospital and Health Research Institute (IIS-Princesa), Madrid, Spain; 2 Immunology Department, La Princesa University Hospital (IIS-Princesa), Madrid, Spain; 3 Microbiology Department, La Princesa University Hospital (IIS-Princesa), Madrid, Spain; 4 Rheumatology Department, La Princesa University Hospital (IIS-Princesa), Madrid, Spain; 5 Pharmaceutical and Health Sciences Department, Faculty of Pharmacy, San Pablo-CEU University, Boadilla del Monte, Spain; 6 Methodology Department, La Princesa University Hospital (IIS-Princesa), Madrid, Spain; 7 Internal Medicine Department, La Princesa University Hospital (IIS-Princesa), Madrid, Spain; 8 Hematology Department, Gregorio Marañón General University Hospital and Health Research Institute (IiSGM), Madrid, Spain; 9 Medicine Department, Faculty of Medicine, Autónoma University, Madrid, Spain

**Keywords:** COVID-19, SARS-CoV-2, longitudinal study, severity, *TLR7*, *TYK2*, *OAS1*, viremia

## Abstract

**Background:**

The host response to SARS-CoV-2 depends on multiple factors including age, gender, underlying diseases and genetic background. Viral sensing and activation of the interferon signaling pathway in the early antiviral response could affect disease outcome. This study evaluated gene expression of Toll-like receptor 7 (*TLR7*), tyrosine kinase 2 (*TYK2*) and 2’-5’ oligoadenylate synthetase 1 (*OAS1*) in two early longitudinal samples from mild, moderate and severe cases.

**Methods:**

Demographic and clinical variables were obtained from 157 COVID-19 patients. Peripheral blood mononuclear cells were used for genotyping and gene expression determination, and plasma for viremia detection, by qPCR and digital PCR, respectively. Gene expression was analyzed using Generalized Linear Mixed Models nested by patient. First, univariate analyses determined which variables were significantly associated with gene expression. Next, multivariate models were built with these variables, following the backward method.

**Results:**

*TLR7* levels were higher in patients with mild disease compared to those with moderate disease and decreased over time. In contrast, patients with severe disease had lower *TLR7* expression, which remained stable. *OAS1* behavior was similar, whereas *TYK2* remained unchanged. Multivariate analysis confirmed the relation of low *TLR7* expression with severity and viremia. *OAS1* levels directly correlated with higher viremia. *TYK2* was associated with *OAS1* expression, and *OAS1* with both *TLR7* and *TYK2* levels.

**Conclusions:**

Low *TLR7* levels were associated with severe COVID-19, and together with *OAS1* expression were modulated over time according to severity. While *TLR7* expression decreased across all severity groups as the disease resolved, *OAS1* expression persisted in severe cases with higher viremia.

## 1 Introduction

SARS-CoV-2 disease (COVID-19) exhibits a variety of clinical manifestations, ranging from asymptomatic infection to severe forms. While mild cases can experience minor symptoms, moderate cases may present with compromised respiratory systems (1), and severe cases are associated with acute pneumonia and organ failure (2). COVID-19 severity outcome is related to risk factors and preexisting conditions, as well as to treatments received during hospitalization. Males have a higher tendency to develop severe forms of COVID-19 compared to females, and the severity risk increases with age (3, 4), obesity (5), and hypertension (6). Our group and others have shown that relevant SARS-CoV-2 RNA levels in peripheral blood, also known as viremia, are associated with poor disease outcome (7–9). Furthermore, several protective factors help lower the severity risk. For example, glucocorticoid or immunosuppressant treatments in hospitalized patients modulate disease response and critical patient evolution (10, 11).

Virus sensing, signal transduction and antiviral effector functions are essential events in the response to SARS-CoV-2. Among these processes, the proteolytic cleavage of the SARS-CoV-2 Spike (S) protein by serine protease 2 (TMPRSS2) facilitates viral endocytosis through the interaction of S protein with the viral cellular receptor angiotensin-converting enzyme 2 (ACE-2) (12). In turn, host cells recognize the viral single-stranded RNA through the Toll-like endosomal receptor 7 (TLR7), inducing type I and III interferon (IFN-I/-III) production (13–15). IFN-I and -III bind to their receptors (IFNAR and IFNLR1/IL10R2, respectively) and activate a signaling cascade that promotes the expression of antiviral resistance-related genes (16, 17). Among them, genes of the 2’-5’-oligoadenylate synthetase (OAS) family activate endoribonuclease L (RNase L), leading to viral RNA degradation (18).

Genome-wide association studies (GWAS) have identified genetic variants associated with COVID-19 severity. These include deficiencies in the X-linked *TLR7* that render men more susceptible to severe COVID-19 (19–23), *IFNAR* (24–26), tyrosine kinase 2 (*TYK2*) (27) and *OAS1* (28, 29). In addition, single nucleotide polymorphisms (SNPs) contribute to disease severity (30). Previous work of our group has validated severity associated SNPs in *TMPRSS2*, showing that rs75603675 is a severity predictor whereas rs713400 is associated with viremia and a poor prognosis (31, 32). Also, *TLR7* rs3853839-GG, *TYK2* rs280500-AG and *OAS1* rs1131454-AA are associated with severe disease (33).

In addition to genetic background, gene expression impairment might have negative consequences for the patient’s outcome. Although it is known that changes in several genes are associated with severe COVID-19 (34), their modulation over time remains incompletely understood. To assess the contribution of *TLR7*, *TYK2* and *OAS1* expression to COVID-19 progression over time, we conducted a longitudinal study on the expression of these three genes in patients with COVID-19. Most of the published works are based on cross-sectional studies of gene expression with data taken at a single time point, although additional longitudinal research has been conducted on long-COVID patients (35). Here, we present a longitudinal study with samples serially collected in the early stages of the disease, which represents a novel contribution compared to that provided by the previously described studies.

This study highlights the role of *TLR7* and *OAS1* expression in COVID-19, revealing associations with viral load and severity. *TLR7* and *OAS1* expression was modulated over time and with severity. Low *TLR7* and high *OAS1* expression were independently associated with viremia, and *TLR7*, *TYK2* and *OAS1* levels were independently associated with one another. These insights contribute to understanding the mechanisms that shape the immune response in COVID-19.

## 2 Methods

### 2.1 Study population and design

This work is a secondary analysis of samples from a retrospective observational study where 1350 patients with COVID-19 treated in La Princesa University Hospital between March 2020 and December 2021, before the vaccination campaign started in Spain, were recruited (33). Inclusion criteria were: SARS-CoV-2 infection confirmed by RT-PCR or antigen/serum testing, age older than 18 years and informed consent, plus availability of two sequential peripheral blood samples collected at different times during the course of the disease. Under these conditions, 120 patients of the previous work were selected for the current longitudinal study. In order to increase the population size, 37 additional patients fulfilling identical admission criteria and hospitalized in the University Hospital la Princesa were included. In total, we assessed 157 patients and 314 blood samples collected over 2 longitudinal time-points (median: 6 days [IQR 5–7 days]). A schematic representation of the workflow can be found in **Supplementary Figure S1**.

### 2.2 Variables

All the variables collected in this study are listed in **Supplementary Table S1**, and can be classified in the following main groups: demographic data, comorbidities, medical treatment administered before blood sample collection (48h before extraction, increasing to 7 days for special treatments such as immunosuppressants), laboratory findings for both blood samples, COVID-19 severity and viremia, gene expression (*TLR7*, *TYK2* and *OAS1*) and SNP determination.

COVID-19 severity was classified at both extraction times as mild, moderate or severe based on a modified version of the 8-point World Health Organization (WHO) Ordinal Scale (WOS) for Clinical Improvement (**Supplementary Table S2**).

Demographic data, comorbidities, treatment and laboratory findings were collected from medical records and included in a codified database removing all identifiable information (Zenodo accession number: 17639534).

### 2.3 DNA extraction, genotyping and SNP selection

Total DNA was extracted from 0.5 mL of peripheral blood using MagNA Pure 2.0 and MagNA Pure LC DNA Isolation Kit (Roche Life Science, Basel, Switzerland). Purified DNA concentration was determined with NanoDrop ND-1000 (Thermo Fisher Scientific, Waltham, MA, USA). Of the 157 patients included in this study, 120 patients from the previous cohort were genotyped as described (33). The additional 37 patients were genotyped by qPCR using predesigned SNP Genotyping Taqman™ Assays (Applied Biosystems) on a CFX384 Touch Real-Time PCR System. Allelic discrimination was determined using the software CFX 3.1 Manager (BioRad, Hercules, CA, USA). Both genotyping strategies were carried on duplicate and included negative controls.

A total of 21 SNPs were analyzed in the previous study (33). Herein, we selected those which retained their association with COVID-19 severity in the multivariate model (*TLR7* rs3853839, *TYK2* rs280500 and rs280519, and *OAS1* rs1131454).

### 2.4 RNA extraction and quantification by RT-PCR

Total RNA was extracted from peripheral blood mononuclear cells (PBMCs) isolated from 10 mL. After erythrocyte lysis, a density gradient was performed using Lymphoprep™ (Cedarlane Laboratories, Burlington, ON, Canada), and cell pellets were resuspended in 1 mL of TRIsure™ (Bioline, London, UK) and processed according to manufacturer’s protocol. A total of 2 µg of cDNA were synthesized using the SuperScript^®^ VILO™ cDNA Synthesis Kit (Thermo Fisher Scientific).

*TLR7, TYK2* and *OAS1* expression was determined by qPCR using TaqMan™ Fast Advanced Master Mix (Applied Biosystems) on a CFX384 Touch Real-Time PCR System (BioRad), following the manufacturer’s recommendations. Predesigned TaqMan™ assays used were: Hs00152971_m1 (*TLR7*), Hs00177464_m1 (*TYK2*), Hs00973635_m1 (*OAS1*), and Hs02800695_m1 (*HPRT*) and Hs00427620_m1 (*TBP*) as housekeeping genes. Each sample was evaluated as a triplicate. Relative mRNA levels were calculated by the 2^-ΔCt^ method, and normalization was carried out using *HPRT* expression due to heterogeneous *TBP* levels across severity groups.

### 2.5 SARS-CoV-2 viral load determination by digital PCR

Viral RNA was extracted in an automated EMAG^®^ platform (BioMérieux, Marcy-l’Étoile, France) from 400 µL of plasma obtained by centrifuging EDTA-treated peripheral blood and eluted in 60 µL.

SARS-CoV-2 viremia determination was performed by QuantStudio Absolute Q digital PCR (Applied Biosystems, Thermo Fisher Scientific, Waltham, MA, USA) using the TaqPath™ COVID-19 CE-IVD RT-PCR Kit (Applied Biosystems), adapted for use with the TaqPath™ one Step digital PCR Kit (Applied Biosystems). Quantification of each gene, ORF, N, and S, was calculated using QuantStudio Absolute Q software. Viral load was determined by the average of the quantification of the three genes amplified.

### 2.6 Statistical analysis

Statistical analyses were conducted using R version 4.4.0.

Quantitative variables with gaussian distribution were expressed as mean and standard deviation (SD), and those with non-normal distribution as median and interquartile range (IQR). Kruskal-Wallis test was used to analyze significant differences. Qualitative variables were described as total counts with frequencies, and Fisher’s exact test or Pearson’s χ^2^ test were used to compare categorical variables.

*TLR7*, *TYK2* and *OAS1* expressions were analyzed using generalized linear mixed models nested by patient with the AR1 variance-covariance matrix to correct for the temporal autocorrelation of repeated measurements over time (glmmTMB package (36)). The distribution that best fitted the expression values of each gene was the log-normal distribution, as estimated by the AIC criterion (rriskDistributions package (37)). First, univariate analyses were performed to determine which demographic, clinical and genetic variables were associated with gene expression, testing all available variables individually. Next, multivariate models were built to better understand the dependence of gene expressions on the set of significant variables identified in the univariate analyses (p<0.10), following the backward method. In the multivariate analyses, variables with more than 15% missing data were excluded. Therefore, no imputations were performed, because the sample size was not sufficiently large. Standardization was applied for comparison of effects on the dependent variable.

The emergency situation of the pandemic resulted in differing time intervals between the first and second extractions among patients; therefore, in all analyses, the variable “days from symptom onset to extraction date” was included to correct for the potential effect of the disease duration in each patient. Similarly, to account for interplate variability, the variable “plate” was included in all analyses.

## 3 Results

### 3.1 Sociodemographic and clinical variables in the study population

The study population included a total of 157 patients, 52.2% of them were male and the mean age was 69.55 years (SD 15.52). The most frequent comorbidities in the population were hypertension, diabetes mellitus and obesity (**Table 1**).

**Table 1:** Demographic data and comorbidities of the study population.

Variable	N	%
Sex		
Male	82	52
Female	75	48
Age (years)		
<45	9	5.7
45-70	68	43
>70	80	51
Hypertension	84	54
Diabetes mellitus	31	20
Obesity	23	15
Dementia	6	3.8
People living with HIV	1	0.6

A descriptive analysis of treatments and laboratory findings for each extraction can be found in **Supplementary Tables S3 and S4**. The variables that showed significant differences among severity groups in both extractions were: treatment with methylprednisolone, immunosuppressants and tocilizumab, and levels of D-dimer, lactate dehydrogenase, C-reactive protein and ferritin. Specifically, at the first extraction, significant variations across severity groups were observed in the *OAS1* rs1131454 genotype, *TLR7* expression, the number of days from symptom onset to extraction date, treatment with glucocorticoids and fibrinogen levels (**Supplementary Table S3**). In the second extraction, significant differences between severity groups were observed for the *TYK2* rs280519 genotype, *TLR7* and *OAS1* levels, and treatment with dexamethasone, viremia and lymphocyte levels (**Supplementary Table S4**).

**Figure 1:**
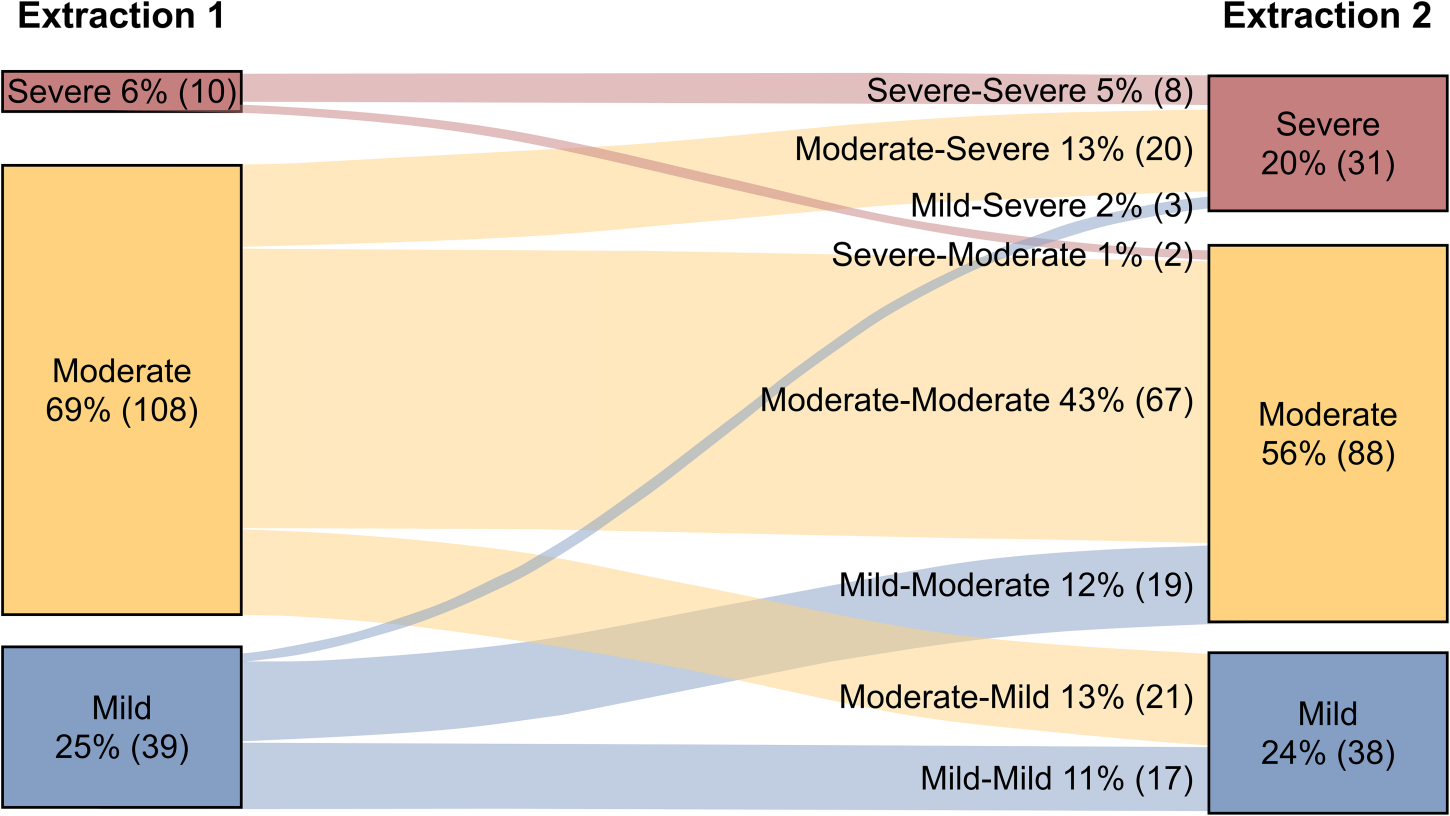
COVID-19 severity evolution. Sankey diagram showing the evolution of severity status among patients, between the first (left boxes) and the second (right boxes) extraction. The bandwidths between both extractions represent the status evolution with lane sizes proportional to the percentage of patients. The number of patients are shown in parentheses.

Considering the longitudinal nature of this study, we took into account the progression of the disease, grouping patients according to their disease severity assessed at each extraction using the WHO severity scale. We then elaborated a Sankey diagram depicting the transitions within severity groups between both extractions. At the initial extraction, 24.8% of patients were classified as having mild disease, 68.8% as having moderate disease, and 6.4% as having severe disease. At the second extraction, the proportion of patients with mild and moderate disease decreased to 24.2% and 56% respectively, while the severe disease percentage increased to 19.7% (**Figure 1**). Thus, a total of 23 patients improved their condition and 42 worsened, whereas 92 maintained the same COVID-19 severity at both extractions.

### 3.2 *TLR7* and *OAS1* expression varies with COVID-19 severity and shows a time-dependent reduction

In order to determine which variables were associated with gene expression, univariate analyses were performed for each gene, adjusting by plate number and days from symptom onset to extraction date. The association between *TLR7* and *OAS1* expression with COVID-19 severity was especially relevant among the significant results obtained (**Supplementary Table S5**). *TLR7* levels showed a decreasing trend with increased COVID-19 severity, being lower in patients with moderate disease (β-coeff: -0.45 [95%CI -0.65, -0.25], p<0.001) and further reduced in patients with severe disease (β-coeff: -0.69 [95%CI -0.99, -0.40], p<0.001) compared to those with mild disease.

Another interesting variable collected in our study was viremia, a known factor associated with COVID-19 severity. In our cohort, patients with severe COVID-19 exhibited higher viremia than those with mild or moderate disease at the first extraction, and these elevated levels persisted at the second sampling (**Supplementary Figure S2**).When evaluating the effect of viremia levels in gene expression, a negative correlation was found between viremia and *TLR7* levels, while there was a tendency for *OAS1* to show increased expression in patients with higher viremia (**Supplementary Table S5**).

**Figure 2:**
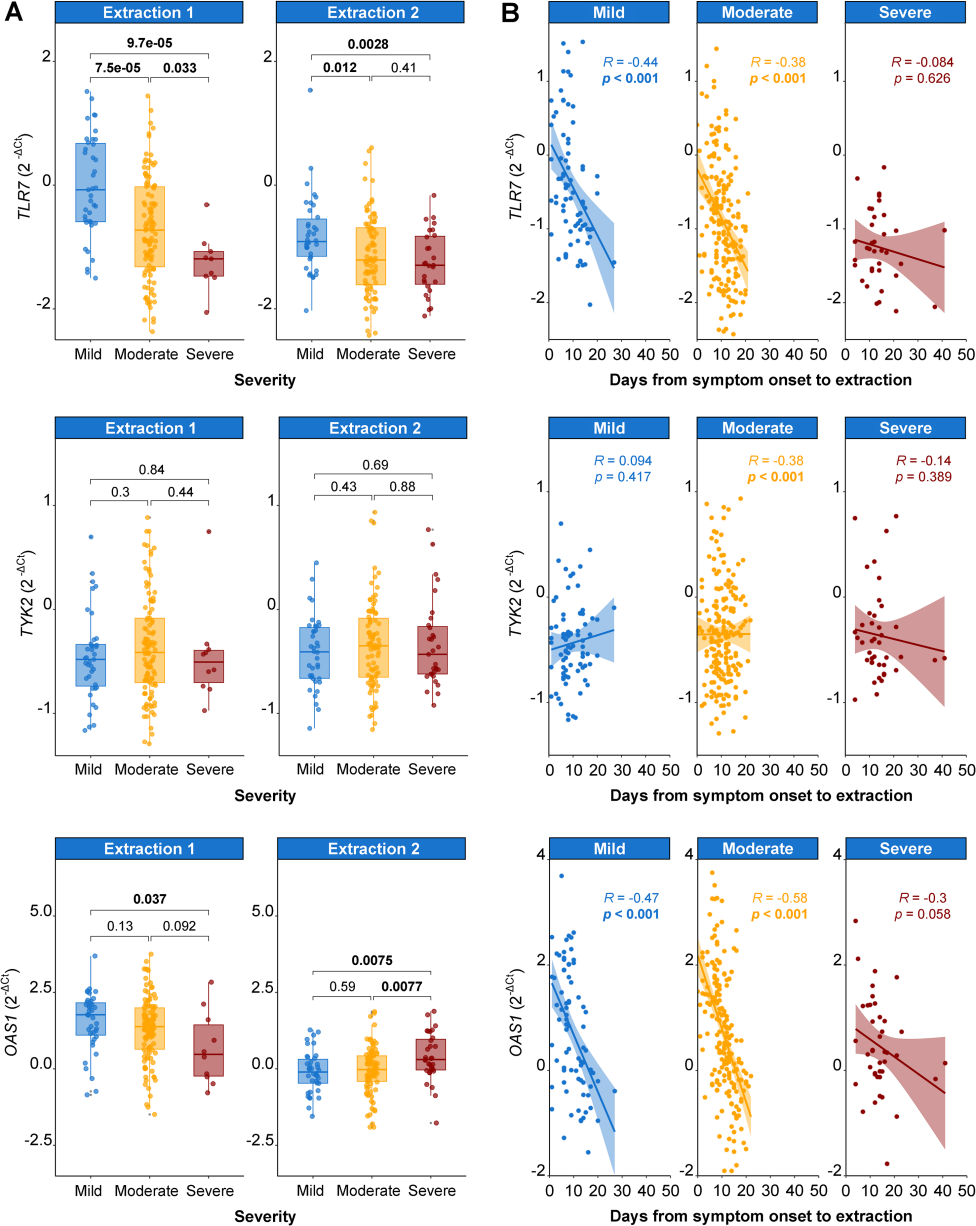
Gene expression modulation according to COVID-19 severity groups **(A)** and over time **(B)**. Data adjusted for plate number and days from symptom onset to extraction date. **(A)***TLR7*, *TYK2* and *OAS1* expression grouped by COVID-19 severity (mild [blue], moderate [yellow] and severe [red]) for each extraction. The boxes represent the median (line) with interquartile range. Statistics: Kruskal-Wallis and Dunn test. **(B)** Linear regression of *TLR7*, *TYK2* and *OAS1* expression over days from symptom onset to extraction date, grouped by severity levels reported at extraction time. Bold: significant *p*-values.

When gene expression was represented across severity groups, the differences in *TLR7* levels remained significant at both extractions (**Figure 2A**, upper panel). On the other hand, *TYK2* levels were not associated with COVID-19 severity (**Figure 2A**, middle panel). However, *OAS1* behaved differently over time within each severity group (**Figure 2A**, lower panel). Patients with mild and moderate disease showed higher *OAS1* levels at the first extraction concurrent with higher viremia, and subsequently reduced its expression at the second extraction, corresponding to a decline in detectable viremia (**Supplementary Figure S2**). In contrast, patients with severe disease were likely unable to sufficiently upregulate *OAS1* to control their higher and more persistent viremia.

Interestingly, time remained significant in all the univariate analyses for *TLR7* and *OAS1* expression (data not shown). To clarify this modulation, gene expression measured in both extractions was plotted against days from symptom onset to each extraction date across COVID-19 severity levels reached at both extractions (**Figure 2B**). There was a downward modulation of *TLR7* and *OAS1* gene expression in patients with mild and moderate disease over time, while levels in patients with severe disease remained stable. Notably, *TLR7* levels in the second extraction remained higher in patients with mild compared to moderate and severe disease. On the contrary, *OAS1* levels in the second extraction were suppressed in patients with mild and moderate compared to severe disease. As previously stated, *TYK2* expression remained unchanged.

### 3.3 Multivariate models reveal associations of *TLR7*, *TYK2* and *OAS1* expression with disease severity, viremia and host factors in COVID-19 patients

**Figure 3:**
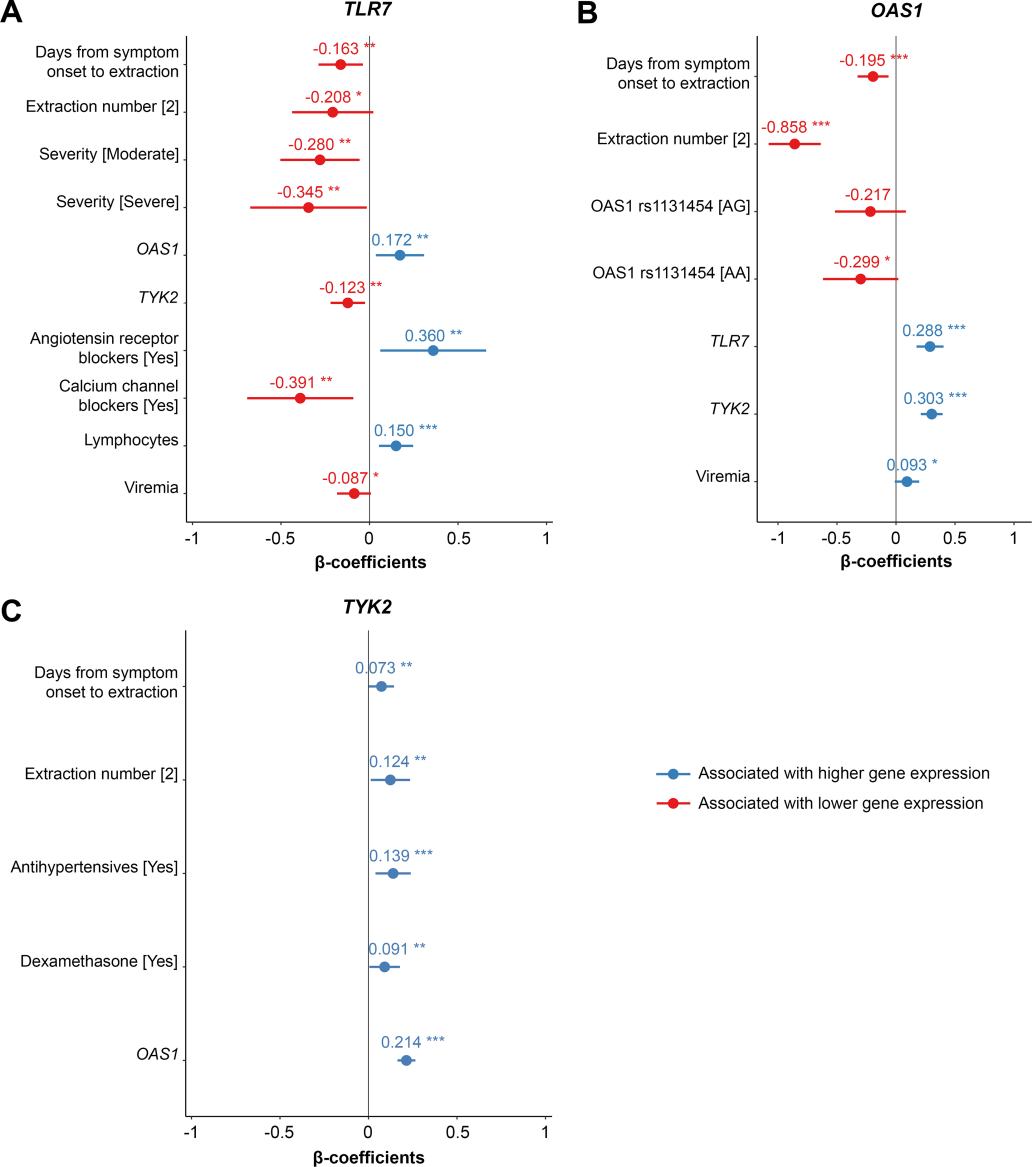
Forest plots of the multivariate analysis of *TLR7***(A)**, *OAS1***(B)** and *TYK2***(C)** expression. The multivariate model was adjusted for plate number and days from symptom onset to extraction date. The plot represents beta-coefficient (dots and numbers) with 95% confidence intervals (horizontal bars) for the significant variables in the model. *p ≤ 0.1; **p ≤ 0.05; ***p ≤ 0.01.

In order to better understand *TLR7*, *TYK2* and *OAS1* effect on SARS-CoV-2 infection, we fitted multivariate analyses for each gene expression including all the variables that were significant in the univariate analyses, obtaining the final models following the backward method, and adjusting for plate number, extraction and days from symptom onset to extraction date (**Figure 3**).

*TLR7* expression was reduced in patients with moderate (β-coeff: -0.28 [95%CI -0.504, -0.056], p=0.014) and severe (β-coeff: -0.345 [95%CI -0.674, -0.015], p=0.04) disease compared to patients with mild disease. Patients with high *TLR7* expression also showed higher *OAS1* levels (β-coeff: 0.172 [95%CI 0.035, 0.308], p=0.014) and lower *TYK2* levels (β-coeff: -0.123 [95%CI -0.220, -0.025], p=0.013). Additionally, angiotensin receptor blocker (ARB) treatment (β-coeff: 0.360 [95%CI 0.061, 0.659], p=0.018) and higher lymphocyte count (β-coeff: 0.15 [95%CI 0.053, 0.247], p=0.002) were associated with greater *TLR7* levels. Calcium channel blocker administration (β-coeff: -0.391 [95%CI -0.691, -0.092], p=0.011) and higher viremia (β-coeff: -0.087 [95%CI -0.184, 0.011], p=0.081) were negatively correlated with *TLR7* expression (**Figure 3A**).

Regarding *OAS1*, a relationship was found between decreased expression and the rs1131454-AA genotype (β-coeff: -0.299 [95%CI -0.62, -0.02], p=0.066). In addition, a positive correlation was detected between *OAS1* expression and both *TLR7* (β-coeff: 0.288 [95%CI 0.17, 0.40], p<0.001) and *TYK2* expression (β-coeff: 0.303 [95%CI 0.21, 0.40], p<0.001). In contrast to *TLR7*, viremia was higher in patients expressing high *OAS1* levels (β-coeff: 0.093 [95%CI -0.01, 0.20], p=0.077) (**Figure 3B**).

Although *TYK2* expression was not associated with severity, we found a positive correlation between *TYK2* and *OAS1* levels (β-coeff: 0.214 [95%CI 0.164, 0.265], *p* < 0.001), adjusted by antihypertensive and dexamethasone treatments (**Figure 3C**).

## 4 Discussion

This longitudinal study has assessed the expression of *TYK2*, *TLR7* and *OAS1* in PBMCs from SARS-CoV-2 infected patients, identifying significant associations between gene expression, severity, viremia and patient biochemical parameters.

In our cohort, patients with moderate and severe disease showed lower *TLR7* expression than those with mild disease at the time of the first extraction. Low *TLR7* expression has been described to be associated with COVID-19 severity (35, 38), and bronchoalveolar lavage fluids from severe and deceased patients have been reported to contain fewer TLR7-expressing cells than mild and samples from survivor patients, respectively (39, 40). However, these investigations were carried out with samples taken at a single time point. In our study we showed a *TLR7* downmodulation at the time of the second extraction in mild and moderate cases. To the best of our knowledge, this is the first longitudinal study focused on investigating the relationship between *TLR7* expression during the acute COVID-19 phase and disease outcome.

**Figure 4:**
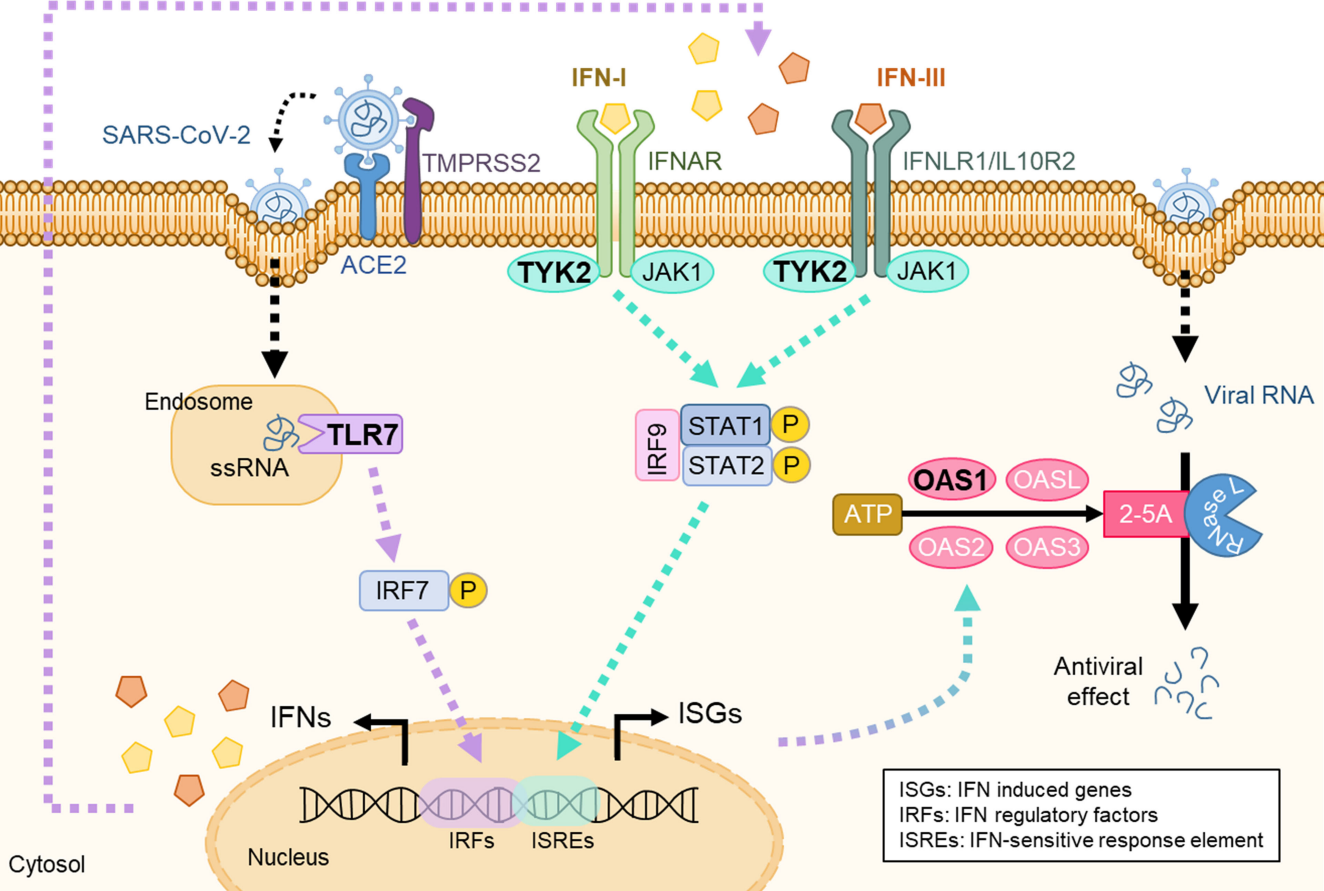
Diagram of virus entry into the cell and activation of the IFN-I and -III signaling pathways. Violet: TLR7 signaling pathway; turquoise: IFN signaling cascade. Bold: genes analyzed in this study.

Regarding the downregulation of *TLR7* expression over time, our results suggest that high *TLR7* levels protect against severe COVID-19 outcome, and as the disease resolves, *TLR7* expression decreases. These data are in agreement with the observation that *TLR7* levels in PBMCs from healthy donors are lower than those in COVID-19 patients (41). Furthermore, in mouse models, the early phase of SARS-CoV-2 infection causes an upregulation of the *Tlr7*, *Irf7* and IFN-I pathways in the lungs, whereas *Tlr7* and *Irf7*-deficient mice show increased COVID-19 severity caused by deficient IFN-I and -III activation (42, 43). Based on these results, we can speculate that patients with mild disease, and to a lesser extent in patients with moderate disease, are able to induce *TLR7* in early infection stages, which triggers the transcription of genes involved in the antiviral response. In contrast, patients unable to induce *TLR7* in early infection stages may develop a severe disease (**Figure 4**).

In our analysis, lower *TLR7* expression was independently associated with both lymphopenia and severe COVID-19, supporting the link between these observations. In accordance with this, low lymphocyte count on admission has been associated with poor COVID-19 outcome, including ICU admission and acute respiratory distress syndrome (44). Several variables related to blood pressure were associated with the expression of *TLR7* and *TYK2* in the multivariate analysis. The effect of antihypertensives on *TYK2* expression could be related to the fact that hypertensive patients have increased activation of the renin-angiotensin-aldosterone system, and angiotensin II activates *TYK2* and *JAK2* through its AT1 receptor (45). On the other hand, there is scarce evidence of any relationship of *TLR7* with ARB and calcium-channel blockers.

We found a tendency for negative correlation between *TLR7* levels and viremia. *TLR7* levels could be a major factor in viral clearance, and patients with impaired *TLR7* expression might be unable to achieve optimal viral elimination. This association is described in SARS-CoV-2 infected mice, where Tlr7 deficiency leads to higher lung viral load 6–10 days post-infection (43).

*TYK2* expression was not associated with COVID-19 severity in our cohort, and its levels were maintained over time. Evidence to date shows controversy about the role of *TYK2* in COVID-19 severity, reporting association with its overexpression (34, 46) or its deficiency (47). Additionally, Akbari et al. stated that, although *TYK2* expression was suppressed in Iranian COVID-19 patients compared to controls, *TYK2* levels could not differentiate properly between non-ICU and ICU admitted patients (48). We have found a positive correlation between *TYK2* and *OAS1* expression that could be explained by the downstream localization of *OAS1* in the IFN-I signaling pathway (49).

Low *OAS1* levels have been associated with higher risk of severe COVID-19 (50, 51). In line with these findings, there was a trend in our population in which patients with mild disease had higher *OAS1* levels than those with severe disease at the first extraction. However, as the infection resolved, *OAS1* was downregulated in patients with mild and moderate disease. Meanwhile, *OAS1* expression was maintained in severe cases, indicating a reactive state. Also, *OAS1* levels were directly related to viremia, consistent with higher *OAS1* expression over time in severe cases.

Furthermore, we detected an association between the rs1131454-AA genotype and a lower *OAS1* expression, in agreement with previous results showing the abundance of a transcript with a premature termination codon (28). These authors have shown that loss of genetically regulated *OAS1* expression impairs spontaneous clearance of SARS-CoV-2 and increases the risk of hospitalization.

In the multivariate analyses we found relations between *TYK2*, *TLR7* and *OAS1* expressions. High *TLR7* and *TYK2* levels correlated with increased *OAS1* expression, and higher *TLR7* was associated with low *TYK2* levels. These data suggest that SARS-CoV-2 infection increases *TLR7* expression in mild and moderate cases, leading to *OAS1* transcription (52) either via IRF-7 binding to DNA or via TYK2-mediated IFN-I signaling pathway (**Figure 4**).

In conclusion, low *TLR7* expression correlates with worse outcome and high viremia in COVID-19 patients. *TLR7* and *OAS1* levels are higher in patients with mild and moderate disease and decrease as disease resolves. In severe cases, *OAS1* expression persists. These findings improve our understanding of the molecular mechanisms involved in the early immune response in COVID-19.

## Data Availability

The original contributions presented in the study are included in the article/**Supplementary Material**, further inquiries can be directed to the corresponding author/s.

## References

[1] Ruan Q Yang K Wang W Jiang L Song J . Clinical predictors of mortality due to COVID-19 based on an analysis of data of 150 patients from Wuhan, China. Intensive Care Med. (2020) 46:846–8. doi: 10.1007/s00134-020-05991-x, PMID: 32125452 PMC7080116

[2] Berlin DA Gulick RM Martinez FJ . Severe covid-19. N Engl J Med. (2020) 383:2451–60. doi: 10.1056/NEJMcp2009575, PMID: 32412710

[3] Jin JM Bai P He W Wu F Liu XF Han DM . Gender differences in patients with COVID-19: focus on severity and mortality. Front Public Health. (2020) 8:152. doi: 10.3389/fpubh.2020.00152, PMID: 32411652 PMC7201103

[4] Starke KR Reissig D Petereit-Haack G Schmauder S Nienhaus A Seidler A . The isolated effect of age on the risk of COVID-19 severe outcomes: a systematic review with meta-analysis. BMJ Glob Health. (2021) 6:e006434. doi: 10.1101/2021.05.27.21257909, PMID: 34916273 PMC8678541

[5] Gao F Zheng KI Wang XB Sun QF Pan KH Wang TY . Obesity is a risk factor for greater COVID-19 severity. Diabetes Care. (2020) 43:e72–4. doi: 10.2337/dc20-0682, PMID: 32409499

[6] Savoia C Volpe M Kreutz R . Hypertension, a moving target in COVID-19: current views and perspectives. Circ Res. (2021) 128:1062–79. doi: 10.1161/CIRCRESAHA.121.318054, PMID: 33793331 PMC8011346

[7] Cardeñoso Domingo L Roy Vallejo E Zurita Cruz ND Chicot Llano M Ávalos Pérez-Urria E Barrios A . Relevant SARS-CoV-2 viremia is associated with COVID-19 severity: Prospective cohort study and validation cohort. J Med Virol. (2022) 94:5260–70. doi: 10.1002/jmv.27989, PMID: 35811284 PMC9349374

[8] Fajnzylber J Regan J Coxen K Corry H Wong C Rosenthal A . SARS-CoV-2 viral load is associated with increased disease severity and mortality. Nat Commun. (2020) 11:5493. doi: 10.1038/s41467-020-19057-5, PMID: 33127906 PMC7603483

[9] Jacobs JL Bain W Naqvi A Staines B Castanha PMS Yang H . Severe acute respiratory syndrome coronavirus 2 viremia is associated with coronavirus disease 2019 severity and predicts clinical outcomes. Clin Infect Dis. (2021) 74:1525. doi: 10.1093/cid/ciab686, PMID: 34374761 PMC9070832

[10] Rosas IO Bräu N Waters M Go RC Hunter BD Bhagani S . Tocilizumab in hospitalized patients with severe covid-19 pneumonia. N Engl J Med. (2021) 384:1503–16. doi: 10.1056/NEJMoa2028700, PMID: 33631066 PMC7953459

[11] Schoot TS Kerckhoffs APM Hilbrands LB van Marum RJ . Immunosuppressive drugs and COVID-19: A review. Front Pharmacol. (2020) 11:1333. doi: 10.3389/fphar.2020.01333, PMID: 32982743 PMC7485413

[12] Hoffmann M Kleine-Weber H Schroeder S Krüger N Herrler T Erichsen S . SARS-coV-2 cell entry depends on ACE2 and TMPRSS2 and is blocked by a clinically proven protease inhibitor. Cell. (2020) 181:271–280.e8. doi: 10.1016/j.cell.2020.02.052, PMID: 32142651 PMC7102627

[13] Diebold SS Kaisho T Hemmi H Akira S Reis e Sousa C . Innate antiviral responses by means of TLR7-mediated recognition of single-stranded RNA. Science. (2004) 303:1529–31. doi: 10.1126/science.1093616, PMID: 14976261

[14] van der Sluis RM Cham LB Gris-Oliver A Gammelgaard KR Pedersen JG Idorn M . TLR2 and TLR7 mediate distinct immunopathological and antiviral plasmacytoid dendritic cell responses to SARS-CoV-2 infection. EMBO J. (2022) 41:e109622. doi: 10.15252/embj.2021109622, PMID: 35178710 PMC9108609

[15] Zhao X Chen D Li X Griffith L Chang J An P . Interferon control of human coronavirus infection and viral evasion: mechanistic insights and implications for antiviral drug and vaccine development. J Mol Biol. (2022) 434:167438. doi: 10.1016/j.jmb.2021.167438, PMID: 34990653 PMC8721920

[16] Le Pen J Rice CM . The antiviral state of the cell: lessons from SARS-CoV-2. Curr Opin Immunol. (2024) 87:102426. doi: 10.1016/j.coi.2024.102426, PMID: 38795501 PMC11260430

[17] Schoggins JW . Interferon-stimulated genes: what do they all do? Annu Rev Virol. (2019) 6:567–84. doi: 10.1146/annurev-virology-092818-015756, PMID: 31283436

[18] Schwartz SL Conn GL . RNA regulation of the antiviral protein 2′-5′-oligoadenylate synthetase. Wiley Interdiscip Rev RNA. (2019) 4):e1534. doi: 10.1002/wrna.1534, PMID: 30989826 PMC6585406

[19] Asano T Boisson B Onodi F Matuozzo D Moncada-Velez M Maglorius Renkilaraj MRL . X-linked recessive TLR7 deficiency in ~1% of men under 60 years old with life-threatening COVID-19. Sci Immunol. (2021) 6:eabl4348. doi: 10.1126/sciimmunol.abl4348, PMID: 34413140 PMC8532080

[20] Fallerini C Daga S Mantovani S Benetti E Picchiotti N Francisci D . Association of Toll-like receptor 7 variants with life-threatening COVID-19 disease in males: findings from a nested case-control study. Elife. (2021) 10:e67569. doi: 10.7554/eLife.67569.sa2, PMID: 33650967 PMC7987337

[21] van der Made CI Simons A Schuurs-Hoeijmakers J van den Heuvel G Mantere T Kersten S . Presence of genetic variants among young men with severe COVID-19. JAMA. (2020) 324:1. doi: 10.1001/jama.2020.13719, PMID: 32706371 PMC7382021

[22] Martínez-Gómez LE Martinez-Armenta C Medina-Luna D Ordoñez-Sánchez ML Tusie-Luna T Ortega-Peña S . Implication of myddosome complex genetic variants in outcome severity of COVID-19 patients. J Microbiol Immunol Infect. (2023) 56:939–50. doi: 10.1016/j.jmii.2023.06.002, PMID: 37365052 PMC10273757

[23] Naushad SM Mandadapu G Ramaiah MJ Almajhdi FN Hussain T . The role of TLR7 agonists in modulating COVID-19 severity in subjects with loss-of-function TLR7 variants. Sci Rep. (2023) 13:13078. doi: 10.1038/s41598-023-40114-8, PMID: 37567916 PMC10421879

[24] Al Qureshah F Le Pen J de Weerd NA Moncada-Velez M Materna M Lin DC . A common form of dominant human IFNAR1 deficiency impairs IFN-α and -ω but not IFN-β-dependent immunity. J Exp Med. (2025) 222:e20241413. doi: 10.1084/jem.20241413, PMID: 39680367 PMC11648951

[25] Zhang Q Bastard P Liu Z Le Pen J Moncada-Velez M Chen J . Inborn errors of type I IFN immunity in patients with life-threatening COVID-19. Science. (2020) 370:eabd4570. doi: 10.1126/science.abd4570, PMID: 32972995 PMC7857407

[26] Zhang Q Matuozzo D Le Pen J Lee D Moens L Asano T . Recessive inborn errors of type I IFN immunity in children with COVID-19 pneumonia. J Exp Med. (2022) 219:e20220131. doi: 10.1084/jem.20220131, PMID: 35708626 PMC9206114

[27] Pairo-Castineira E Rawlik K Bretherick AD Qi T Wu Y Nassiri I . GWAS and meta-analysis identifies 49 genetic variants underlying critical COVID-19. Nature. (2023) 617:764. doi: 10.1038/s41586-023-06034-3, PMID: 37198478 PMC10208981

[28] Banday AR Stanifer ML Florez-Vargas O Onabajo OO Papenberg BW Zahoor MA . Genetic regulation of OAS1 nonsense-mediated decay underlies association with COVID-19 hospitalization in patients of European and African ancestries. Nat Genet. (2022) 54:1103–16. doi: 10.1038/s41588-022-01113-z, PMID: 35835913 PMC9355882

[29] Huffman JE Butler-Laporte G Khan A Pairo-Castineira E Drivas TG Peloso GM . Multi-ancestry fine mapping implicates OAS1 splicing in risk of severe COVID-19. Nat Genet. (2022) 54:125. doi: 10.1038/s41588-021-00996-8, PMID: 35027740 PMC8837537

[30] Colona VL Vasiliou V Watt J Novelli G Reichardt JKV . Update on human genetic susceptibility to COVID-19: susceptibility to virus and response. Hum Genomics. (2021) 15:57. doi: 10.1186/s40246-021-00356-x, PMID: 34429158 PMC8384585

[31] Roy-Vallejo E Fernández De Córdoba-Oñate S Delgado-Wicke P Triguero-Martínez A Montes N Carracedo-Rodríguez R . Occurrence of SARS-CoV-2 viremia is associated with genetic variants of genes related to COVID-19 pathogenesis. Front Med (Lausanne). (2023) 10:1215246. doi: 10.3389/fmed.2023.1215246, PMID: 37809329 PMC10557488

[32] Villapalos-García G Zubiaur P Rivas-Durán R Campos-Norte P Arévalo-Román C Fernández-Rico M . Transmembrane protease serine 2 (TMPRSS2) rs75603675, comorbidity, and sex are the primary predictors of COVID-19 severity. Life Sci Alliance. (2022) 5:e202201396. doi: 10.26508/lsa.202201396, PMID: 35636966 PMC9152129

[33] Delgado-Wicke P Fernández de Córdoba-Oñate S Roy-Vallejo E Alegría-Carrasco E Rodríguez-Serrano DA Lamana A . Genetic variants regulating the immune response improve the prediction of COVID-19 severity provided by clinical variables. Sci Rep. (2024) 14:1–10. doi: 10.1038/s41598-024-71476-2, PMID: 39237611 PMC11377536

[34] Pairo-Castineira E Clohisey S Klaric L Bretherick AD Rawlik K Pasko D . Genetic mechanisms of critical illness in COVID-19. Nature. (2021) 591:92–8. doi: 10.1038/s41586-020-03065-y, PMID: 33307546

[35] Gómez-Carballa A Pardo-Seco J Pischedda S Rivero-Calle I Butler-Laporte G Richards JB . Sex-biased expression of the TLR7 gene in severe COVID-19 patients: Insights from transcriptomics and epigenomics. Environ Res. (2022) 215:114288. doi: 10.1016/j.envres.2022.114288, PMID: 36152884 PMC9508271

[36] Brooks ME Kristensen K van Benthem KJ Magnusson A Berg CW Nielsen A . glmmTMB balances speed and flexibility among packages for zero-inflated generalized linear mixed modeling. R J. (2017) 9:378–400. doi: 10.32614/RJ-2017-066

[37] Belgorodski N Greiner M Tolksdorf K Schueller K . rriskDistributions: fitting distributions to given data or known quantiles (2017). Available online at: https://CRAN.R-project.org/package=rriskDistributions (Accessed September 19, 2025).

[38] Lee N Ko R Lee SY . Differential expression patterns of toll-like receptors in COVID-19 patients. FBL. (2023) 28:307. doi: 10.31083/j.fbl2811307, PMID: 38062845

[39] Sorrentino L Fracella M Frasca F D’Auria A Santinelli L Maddaloni L . Alterations in the expression of IFN lambda, IFN gamma and toll-like receptors in severe COVID-19 patients. Microorganisms. (2023) 11:689. doi: 10.3390/microorganisms11030689, PMID: 36985262 PMC10058642

[40] Wu D Yang XO . Dysregulation of pulmonary responses in severe COVID-19. Viruses. (2021) 13:957. doi: 10.3390/v13060957, PMID: 34064104 PMC8224314

[41] Arefinia N Banafi P Zarezadeh MA Mousawi HS Yaghobi R Farokhnia M . TLR3, TLR7, and TLR8 genes expression datasets in COVID-19 patients: Influences of the disease severity and gender. Data Brief. (2024) 54:110498. doi: 10.1016/j.dib.2024.110498, PMID: 38868379 PMC11166686

[42] Ghimire R Shrestha R Amaradhi R Liu L More S Ganesh T . Toll-like receptor 7 (TLR7)-mediated antiviral response protects mice from lethal SARS-CoV-2 infection. J Virol. (2025) 99:e0166824. doi: 10.1128/jvi.01668-24, PMID: 40162785 PMC12090760

[43] Wang C Khatun MS Ellsworth CR Chen Z Islamuddin M Nisperuza Vidal AK . Deficiency of Tlr7 and Irf7 in mice increases the severity of COVID-19 through the reduced interferon production. Commun Biol. (2024) 7:1162. doi: 10.1038/s42003-024-06872-5, PMID: 39289468 PMC11408513

[44] Huang I Pranata R . Lymphopenia in severe coronavirus disease-2019 (COVID-19): systematic review and meta-analysis. J Intensive Care. (2020) 8:36. doi: 10.1186/s40560-020-00453-4, PMID: 32483488 PMC7245646

[45] Marrero MB Schieffer B Paxton WG Heerdt L Berk BC Delafontaine P . Direct stimulation of Jak/STAT pathway by the angiotensin II AT1 receptor. Nature. (1995) 375:247–50. doi: 10.1038/375247a0, PMID: 7746328

[46] Hadjadj J Yatim N Barnabei L Corneau A Boussier J Smith N . Impaired type I interferon activity and inflammatory responses in severe COVID-19 patients. Science. (2020) 369:718–24. doi: 10.1126/science.abc6027, PMID: 32661059 PMC7402632

[47] Zabihi Rizi F Ghorbani A Zahtab P Darbaghshahi NN Ataee N Pourhamzeh P . TYK2 single-nucleotide variants associated with the severity of COVID-19 disease. Arch Virol. (2023) 168:119. doi: 10.1007/s00705-023-05729-2, PMID: 36959416 PMC10035968

[48] Akbari M Akhavan-Bahabadi M Shafigh N Taheriazam A Hussen BM Sayad A . Expression analysis of IFNAR1 and TYK2 transcripts in COVID-19 patients. Cytokine. (2022) 153:155849. doi: 10.1016/j.cyto.2022.155849, PMID: 35339044 PMC8894869

[49] Silverman RH . A scientific journey through the 2-5A/RNase L system. Cytokine Growth Factor Rev. (2007) 18:381. doi: 10.1016/j.cytogfr.2007.06.012, PMID: 17681844 PMC2075094

[50] Gajate-Arenas M Fricke-Galindo I García-Pérez O Domínguez-de-Barros A Pérez-Rubio G Dorta-Guerra R . The immune response of OAS1, IRF9, and IFI6 genes in the pathogenesis of COVID-19. Int J Mol Sci. (2024) 25:4632. doi: 10.3390/ijms25094632, PMID: 38731851 PMC11083791

[51] Zhou S Butler-Laporte G Nakanishi T Morrison DR Afilalo J Afilalo M . A Neanderthal OAS1 isoform protects individuals of European ancestry against COVID-19 susceptibility and severity. Nat Med. (2021) 27:659–67. doi: 10.1038/s41591-021-01281-1, PMID: 33633408

[52] Yan Q Li P Ye X Huang X Feng B Ji T . Longitudinal peripheral blood transcriptional analysis reveals molecular signatures of disease progression in COVID-19 patients. J Immunol. (2021) 206:2146–59. doi: 10.4049/jimmunol.2001325, PMID: 33846224

