# Supplementary material for: Modulation of TLR7, TYK2 and OAS1 expression during SARS-CoV-2 infection
Source: Front Immunol. 2025 Dec 9;16:1713928. doi: 10.3389/fimmu.2025.1713928 (PMC12722446; doi:10.3389/fimmu.2025.1713928)
Supplement: Supplementary file 1 [file DataSheet1.pdf]

Supplementary Information for:

Modulation of *TLR7*, *TYK2* and *OAS1* expression during SARS-CoV-2 infection

Estíbaliz Alegría-Carrasco[1], Marta Jaén-Castaño[1], Pablo Delgado-Wicke[1,2], Nelly D Zurita-Cruz[3], Nuria Montes[4,5,6], Emilia Roy-Vallejo[7], Sara Fernández de Córdoba-Oñate[4,8], Ana Nicolao-Gómez[1], Rosa Carracedo-Rodríguez[1], Ana Marcos-Jiménez[2], Laura Cardeñoso-Domingo[3], Isidoro González-Álvaro[4,†,*], Elena Fernández-Ruiz[1,9,†,*]

[1]Molecular Biology Unit, La Princesa University Hospital and Health Research Institute (IIS-Princesa), Madrid, Spain

[2]Immunology Department, La Princesa University Hospital (IIS-Princesa), Madrid, Spain

[3]Microbiology Department, La Princesa University Hospital (IIS-Princesa), Madrid, Spain

[4]Rheumatology Department, La Princesa University Hospital (IIS-Princesa), Madrid, Spain

[5]Pharmaceutical and Health Sciences Department, Faculty of Pharmacy, San Pablo-CEU University, Boadilla del Monte, Spain

[6]Methodology Department, La Princesa University Hospital (IIS-Princesa), Madrid, Spain

[7]Internal Medicine Department, La Princesa University Hospital (IIS-Princesa), Madrid, Spain

[8]Hematology Department, Gregorio Marañón General University Hospital and Health Research Institute (IiSGM), Madrid, Spain

[9]Medicine Department, Faculty of Medicine, Autonomous University, Madrid, Spain

[†]These authors contributed equally to this work and share senior authorship.

*Corresponding authors: Isidoro González-Álvaro (isidoro.ga@ser.es) and Elena Fernández-Ruiz (efruiz@salud.madrid.org).

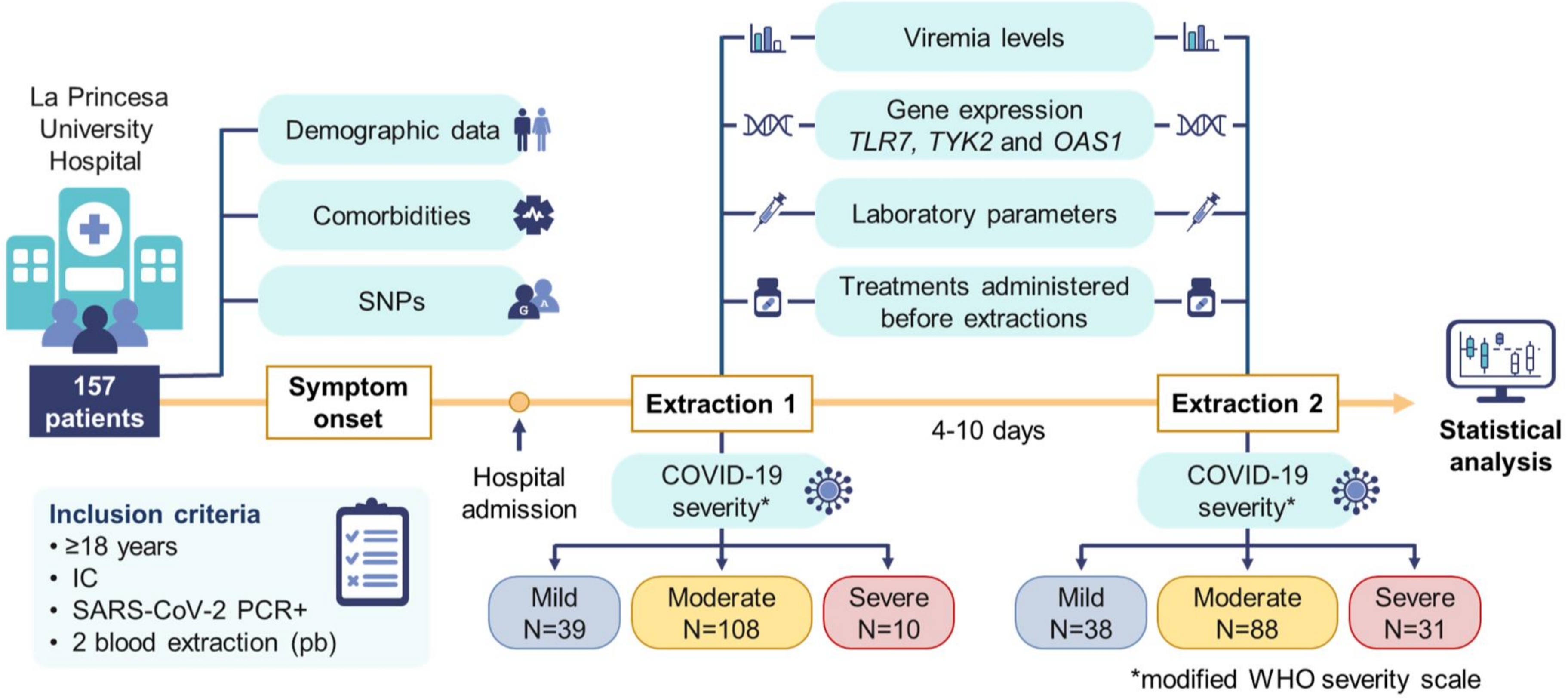

Figure S1. Study workflow. IC: informed consent; pb: peripheral blood; SNP: single nucleotide polymorphism; WHO: World Health Organization.

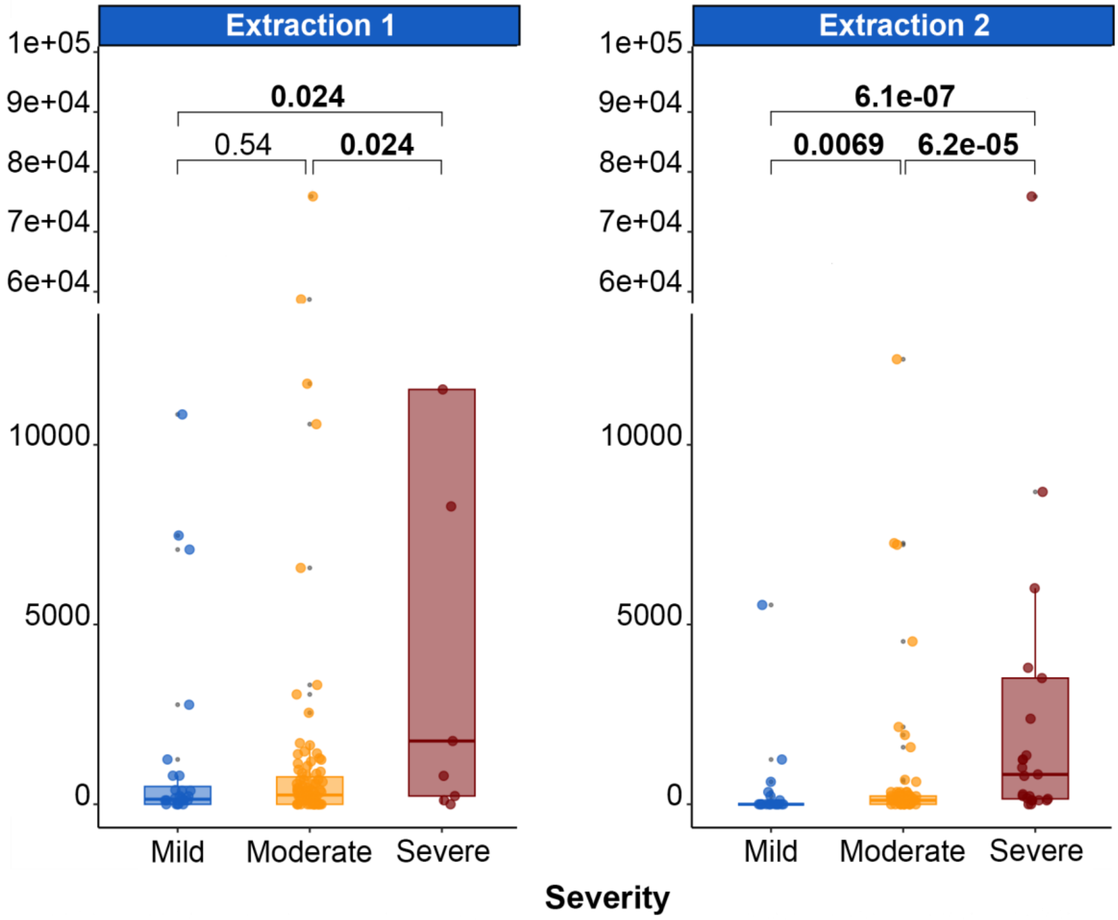

Figure S2. Viral load in COVID-19 patients grouped according to disease severity for each blood extraction. Severity was classified as mild (blue), moderate (yellow) and severe (red). Significant differences are shown between severe compared to mild or moderate disease at the first extraction (left), and between all conditions at the second extraction (right), increasing viral load with the severity. The boxes represent the median (line) with interquartile range. Statistics: Kruskal-Wallis. Bold: significant *p*-values.

Table S1. Baseline variables collected at both extractions during this study. SNP: single nucleotide polymorphism; HIV: human immunodeficiency virus; LDH: lactate dehydrogenase; CRP: C-reactive protein; IL-6: interleukin 6; WHO: World Health Organization.

Variables							
**SNPs**	TYK2 rs280500	TYK2 rs280519	TLR7 rs3853839	OAS1 rs1131454			
**Demographic data and comorbidities**	Sex	Age (categories)					
	Hypertension	Obesity	Dementia	Diabetes mellitus	People living with HIV		
**Previous treatments**	Anticoagulant	Antiplatelet	Hyperimmune plasma				
	Antihypertensive (AH)	AH: angiotensin converting enzyme inhibitor	AH: angiotensin II receptor blocker	AH: diuretics	AH: alpha blockers	AH: beta blockers	AH: calcium channel blockers
	Glucocorticoids (GCC)	GCC: dexamethasone	GCC: methylprednisolone	GCC: others			
	Immunosuppressants (IS)	IS: anakinra	IS: baricitinib	IS: tocilizumab	IS: colchicine	IS: lenalidomide	
	Antivirals (AV)	AV: remdesivir	AV: plitidepsin	AV: entecavir	AV: dovato	AV: tenofovir	AV: aciclovir
**Laboratory data**	Lymphocytes (cells/μL)	Fibrinogen (mg/dL)	D-dimer (μg/mL)	LDH (U/L)	CRP (mg/dL)	Ferritin (ng/mL)	IL-6 (pg/mL)
**Gene expression ($2^{-\Delta Ct}$)**	*TYK2*	*TLR7*	*OAS1*				
**Severity**	WHO scale at extraction						
**Dates**	Date of symptom onset	Extraction date	Time from symptom onset to extraction				
**Extraction number**	Extraction						
**Plate number**	Plate						
**Viremia**	Peripheral blood viral load (copies/mL)						

Table S2. Modified WHO COVID ordinal outcomes scale from the Ordinal Scale for Clinical Improvement. From the World Health Organization. WHO R&D Blueprint. Novel Coronavirus COVID-19 Therapeutic Trial Synopsis, 2020. ECMO: extracorporeal membrane oxygenation; RRT: renal replacement therapy.

Available at: https://www.who.int/publications/i/item/covid-19-therapeutic-trial-synopsis

	Modified WHO scale		Ordinal WHO scale	
**Patient State**	**Descriptor**	**Score**	**Descriptor**	**Score**
**Group 1: mild disease**	No limitation of activities	1	No limitation of activities	1
	Limitation of activities	2	Limitation of activities	2
	Hospitalized, no oxygen therapy	3	Hospitalized, no oxygen therapy	3
**Group 2: moderate disease**	Oxygen by mask or nasal cannula	4	Oxygen by mask or nasal prongs	4
**Group 3: severe disease/death**	Non-invasive ventilation or high-flow oxygen	5	Non-invasive ventilation or high-flow oxygen	5
	Intubation and mechanical ventilation	6	Intubation and mechanical ventilation	6
	Death	7	Ventilation + additional organ support - pressors, RRT, ECMO	7
			Death	8

Table S3. Descriptive analysis of the population during extraction 1, according to COVID-19 severity. ACEI: angiotensin-converting enzyme inhibitors; ARB: angiotensin II receptor blockers; GCC: glucocorticoids; IQR: interquartile range; RR: reference range; LDH: lactate dehydrogenase; CRP: C-reactive protein. [1]: n (%); [2]: Fisher's exact test; Kruskal-Wallis rank sum test; Pearson's Chi-squared test.

Extraction 1	Variable	N	Mild N = 39[1]	Moderate N = 108[1]	Severe N = 10[1]	*p*-value[2]
SNPs genotyping	***TYK2* rs280519**	155 (99%)	39	106 (98%)	10	0.21
	GG	45 (29%)	10 (26%)	30 (28%)	5 (50%)	
	AG	64 (41%)	21 (54%)	41 (39%)	2 (20%)	
	AA	46 (30%)	8 (21%)	35 (33%)	3 (30%)	
	Missing	2	0	2	0	
	***TYK2* rs280500**	157				0.14
	AA	106 (68%)	30 (77%)	68 (63%)	8 (80%)	
	AG	47 (30%)	9 (23%)	37 (34%)	1 (10%)	
	GG	4 (2.5%)	0 (0%)	3 (2.8%)	1 (10%)	
	***OAS1* rs1131454**	157				0.076
	GG	34 (22%)	6 (15%)	24 (22%)	4 (40%)	
	AG	70 (45%)	24 (62%)	44 (41%)	2 (20%)	
	AA	53 (34%)	9 (23%)	40 (37%)	4 (40%)	
	***TLR7* rs3853839**	157				0.49
	CC	112 (71%)	29 (74%)	75 (69%)	8 (80%)	
	CG	28 (18%)	6 (15%)	22 (20%)	0 (0%)	
	GG	17 (11%)	4 (10%)	11 (10%)	2 (20%)	
Gene expression	***TYK2***	157				0.47
	Mean (SD), Median [IQR]	0.762 (0.424), 0.650 [0.485, 0.859]	0.693 (0.344), 0.618 [0.472, 0.719]	0.791 (0.442), 0.661 [0.493, 0.920]	0.725 (0.500), 0.604 [0.478, 0.679]	
	Range	0.274 - 2.421	0.312 - 2.008	0.274 - 2.421	0.378 - 2.114	
	TLR7	157				**<0.001**
	Mean (SD), Median [IQR]	0.836 (0.838), 0.541 [0.303, 1.021]	1.336 (1.078), 0.928 [0.550, 1.991]	0.705 (0.686), 0.481 [0.261, 0.974]	0.293 (0.184), 0.272 [0.225, 0.342]	
	Range	0.038 - 4.543	0.223 - 4.543	0.075 - 4.226	0.038 - 0.728	
	OAS1	157				0.057
	Mean (SD), Median [IQR]	6.147 (6.880), 4.163 [1.906, 7.726]	6.818 (6.644), 5.872 [2.859, 8.891]	6.115 (7.098), 3.981 [1.894, 7.378]	3.880 (5.249), 1.612 [0.764, 4.984]	
	Range	0.201 - 42.599	0.411 - 40.090	0.201 - 42.599	0.449 - 17.055	

Extraction 1	Variable	N	Mild<br>N = 39[1]	Moderate<br>N = 108[1]	Severe<br>N = 10[1]	*p*-value[2]
Housekeeping	**Ct *HPRT***	157				0.98
	Mean (SD),<br>Median [IQR]	25.762 (0.695),<br>25.671 [25.283, 26.056]	25.696 (0.634),<br>25.785 [25.302, 26.001]	25.794 (0.720),<br>25.584 [25.280, 26.160]	25.675 (0.690),<br>25.780 [25.056, 26.016]	
	Range	24.312 - 27.848	24.312 - 27.359	24.727 - 27.848	24.651 - 26.815	
	**Ct *TBP***	157				0.20
	Mean (SD),<br>Median [IQR]	25.747 (0.590),<br>25.685 [25.337, 26.106]	25.691 (0.597),<br>25.542 [25.196, 26.002]	25.743 (0.596),<br>25.672 [25.355, 26.096]	25.999 (0.482),<br>26.051 [25.693, 26.431]	
	Range	24.676 - 27.402	24.766 - 27.234	24.676 - 27.402	25.208 - 26.659	
Time	**Days from symptom onset to extraction**	157				**0.014**
	Mean (SD),<br>Median [IQR]	7.955 (4.364),<br>8.000 [6.000, 10.000]	6.205 (2.957),<br>7.000 [4.000, 8.000]	8.278 (3.729),<br>8.000 [6.000, 10.000]	11.300 (10.045),<br>9.000 [4.000, 14.000]	
	Range	0.000 - 37.000	1.000 - 12.000	0.000 - 20.000	4.000 - 37.000	
Treatments during hospitalization	**Antihypertensives**	60 (38%)	15 (38%)	43 (40%)	2 (20%)	0.55
	ACEI	19 (12%)	7 (18%)	11 (10%)	1 (10%)	0.40
	ARB	17 (11%)	4 (10%)	13 (12%)	0 (0%)	0.75
	Diuretics	22 (14%)	4 (10%)	17 (16%)	1 (10%)	0.79
	Beta-blockers	18 (11%)	5 (13%)	13 (12%)	0 (0%)	0.76
	Calcium channel blockers	16 (10%)	2 (5.1%)	14 (13%)	0 (0%)	0.30
	Alpha blockers	0 (0%)	0 (0%)	0 (0%)	0 (0%)	>0.99
	**Anticoagulants**	118 (75%)	28 (72%)	84 (78%)	6 (60%)	0.34
	**Antiplatelets**	28 (18%)	8 (21%)	19 (18%)	1 (10%)	0.83
	**GCC**	116 (74%)	20 (51%)	87 (81%)	9 (90%)	**0.001**
	Dexamethasone	84 (54%)	17 (44%)	64 (59%)	3 (30%)	0.082
	Methylprednisolone	48 (31%)	2 (5.1%)	39 (36%)	7 (70%)	**<0.001**
	Other GCC	17 (11%)	2 (5.1%)	14 (13%)	1 (10%)	0.37
	**Immunosuppressants**	10 (6.4%)	2 (5.1%)	5 (4.6%)	3 (30%)	**0.027**
	Anakinra	0 (0%)	0 (0%)	0 (0%)	0 (0%)	>0.99
	Tocilizumab	6 (3.8%)	0 (0%)	3 (2.8%)	3 (30%)	**0.003**
	Baricitinib	1 (0.6%)	0 (0%)	1 (0.9%)	0 (0%)	>0.99
	Colchicine	2 (1.3%)	1 (2.6%)	1 (0.9%)	0 (0%)	0.53
	Lenalidomide	1 (0.6%)	1 (2.6%)	0 (0%)	0 (0%)	0.31

Extraction 1	Variable	N	Mild<br>N = 39[1]	Moderate<br>N = 108[1]	Severe<br>N = 10[1]	*p*-value[2]
**Treatments during hospitalization** (cont)	**Antivirals**	4 (2.5%)	2 (5.1%)	2 (1.9%)	0 (0%)	0.45
	Remdesivir	1 (0.6%)	0 (0%)	1 (0.9%)	0 (0%)	>0.99
	Plitidepsin	0 (0%)	0 (0%)	0 (0%)	0 (0%)	>0.99
	Entecavir	2 (1.3%)	2 (5.1%)	0 (0%)	0 (0%)	0.10
	Dovato	1 (0.6%)	0 (0%)	1 (0.9%)	0 (0%)	>0.99
	Tenofovir	0 (0%)	0 (0%)	0 (0%)	0 (0%)	>0.99
	Aciclovir	2 (1.3%)	2 (5.1%)	0 (0%)	0 (0%)	0.10
	**Hyperimmune serum**	0 (0%)	0 (0%)	0 (0%)	0 (0%)	>0.99
**Laboratory parameters**	**Lymphocytes (cells/µL) RR: 1000-4000**	152 (97%)	37 (95%)	105 (97%)	10 (100%)	0.54
	Mean (SD), Median [IQR]	1,151.974 (2,719.626), 880.000 [640.000, 1,165.000]	1,016.216 (496.585), 910.000 [800.000, 1,120.000]	1,219.714 (3,260.297), 850.000 [630.000, 1,160.000]	943.000 (316.405), 1,035.000 [720.000, 1,180.000]	
	Range	250.000 - 34,070.000	340.000 - 2,760.000	250.000 - 34,070.000	480.000 - 1,410.000	
	Missing	5	2	3	0	
	**Fibrinogen (mg/dL) RR: 150-400**	125 (80%)	27 (69%)	90 (83%)	8 (80%)	**<0.001**
	Mean (SD), Median [IQR]	672.896 (149.549), 651.000 [577.000, 744.000]	570.926 (104.159), 577.000 [520.000, 620.000]	697.322 (145.119), 671.500 [601.000, 789.000]	742.250 (186.687), 722.000 [670.500, 856.000]	
	Range	332.000 - 1,098.000	332.000 - 823.000	396.000 - 1,098.000	420.000 - 1,021.000	
	Missing	32	12	18	2	
	**D-Dimer (µg/mL) RR: 0.15-0.5**	131 (83%)	31 (79%)	92 (85%)	8 (80%)	**0.003**
	Mean (SD), Median [IQR]	1.035 (1.546), 0.700 [0.430, 1.060]	0.562 (0.305), 0.460 [0.380, 0.790]	1.133 (1.749), 0.750 [0.450, 1.115]	1.738 (1.591), 0.930 [0.795, 2.390]	
	Range	0.000 - 15.140	0.030 - 1.290	0.000 - 15.140	0.450 - 5.220	
	Missing	26	8	16	2	
	**LDH (U/L) RR: 135-225**	142 (90%)	31 (79%)	104 (96%)	7 (70%)	**<0.001**
	Mean (SD), Median [IQR]	311.162 (134.320), 279.000 [230.000, 355.000]	291.065 (200.325), 231.000 [200.000, 303.000]	305.740 (96.939), 279.000 [236.500, 368.500]	480.714 (160.881), 454.000 [337.000, 632.000]	
	Range	148.000 - 1,283.000	168.000 - 1,283.000	148.000 - 605.000	336.000 - 747.000	
	Missing	15	8	4	3	

Extraction 1	Variable	N	Mild<br>N = 39[1]	Moderate<br>N = 108[1]	Severe<br>N = 10[1]	*p*-value[2]
Laboratory parameters (cont)	**CRP (mg/dL)**<br>**RR: 0-0.5**	154 (98%)	38 (97%)	106 (98%)	10 (100%)	**<0.001**
	Mean (SD),<br>Median [IQR]	7.419 (6.229),<br>5.555 [2.900, 10.130]	4.500 (3.922),<br>3.435 [1.620, 6.940]	7.945 (6.251),<br>5.870 [3.570, 10.820]	12.938 (8.220),<br>13.690 [6.880, 16.200]	
	Range	0.190 - 34.660	0.190 - 15.060	0.210 - 34.660	0.850 - 27.400	
	Missing	3	1	2	0	
	**IL-6 (pg/mL)**<br>**RR: <6**	125 (80%)	24 (62%)	94 (87%)	7 (70%)	0.13
	Mean (SD),<br>Median [IQR]	30.482 (79.735),<br>10.600 [2.000, 24.000]	35.475 (108.707),<br>11.900 [3.000, 21.500]	24.551 (64.815),<br>8.750 [1.800, 24.000]	93.014 (125.557),<br>37.000 [12.000, 141.800]	
	Range	0.000 - 585.000	0.000 - 542.000	0.000 - 585.000	0.000 - 354.200	
	Missing	32	15	14	3	
	**Ferritin (ng/mL)**<br>**RR: 30-400**	134 (85%)	32 (82%)	96 (89%)	6 (60%)	**0.008**
	Mean (SD),<br>Median [IQR]	1,155.328 (2,230.040),<br>633.500<br>[308.000, 1,414.000]	1,385.844 (4,284.489),<br>376.500<br>[257.000, 835.000]	1,034.875 (887.941),<br>670.500<br>[346.500, 1,713.500]	1,853.167 (1,512.652),<br>1,295.000<br>[994.000, 2,093.000]	
	Range	23.000 - 24,519.000	51.000 - 24,519.000	23.000 - 3,782.000	661.000 - 4,781.000	
	Missing	23	7	12	4	
**Viremia**	**Viremia (copies/mL)**	127				0.052
	Mean (SD), Median [IQR]	2,849.961 (10,628.574),<br>230.000<br>[0.000, 850.000]	1,196.667 (2,685.822),<br>140.000<br>[0.000, 595.000]	2,256.204 (10,109.444),<br>257.500<br>[0.000, 790.000]	13,931.111 (21,592.110),<br>1,760.000<br>[230.000, 11,540.000]	
	Range	0.000 - 75,896.667	0.000 - 10,845.000	0.000 - 75,896.667	0.000 - 52,013.333	
	Missing	30	11	18	1	
**Demographic and clinical data**	**Sex**	157				0.89
	Male	82 (52%)	19 (49%)	58 (54%)	5 (50%)	
	Female	75 (48%)	20 (51%)	50 (46%)	5 (50%)	
	**Age (years)**	157				0.20
	<45	9 (5.7%)	3 (7.7%)	5 (4.6%)	1 (10%)	
	45-70	68 (43%)	17 (44%)	44 (41%)	7 (70%)	
	>70	80 (51%)	19 (49%)	59 (55%)	2 (20%)	
	**Hypertension**	84 (54%)	21 (54%)	59 (55%)	4 (40%)	0.71

Extraction 1	Variable	N	Mild<br>N = 39[1]	Moderate<br>N = 108[1]	Severe<br>N = 10[1]	*p*-value[2]
**Demographic and clinical data** (cont)	**Dementia**	6 (3.8%)	3 (7.7%)	3 (2.8%)	0 (0%)	0.35
	**Obesity**	23 (15%)	3 (7.7%)	17 (16%)	3 (30%)	0.17
	**People living with HIV**	1 (0.6%)	0 (0%)	1 (0.9%)	0 (0%)	>0.99
	**Diabetes mellitus**	31 (20%)	7 (18%)	22 (20%)	2 (20%)	0.94

Table S4. Descriptive analysis of the population during extraction 2, according to COVID-19 severity. ACEI: angiotensin-converting enzyme inhibitors; ARB: angiotensin II receptor blockers; GCC: glucocorticoids; IQR: interquartile range; RR: reference range; LDH: lactate dehydrogenase; CRP: C-reactive protein. [1]: n (%); [2]: Fisher's exact test; Kruskal-Wallis rank sum test; Pearson's Chi-squared test.

Extraction 2	Variable	N	Mild<br>N = 38[1]	Moderate<br>N = 88[1]	Severe<br>N = 31[1]	*p*-value[2]
**SNPs genotyping**	***TYK2* rs280519**	155 (99%)	38	86 (98%)	31	**0.021**
	GG	45 (29%)	5 (13%)	26 (30%)	14 (45%)	
	AG	64 (41%)	18 (47%)	39 (45%)	7 (23%)	
	AA	46 (30%)	15 (39%)	21 (24%)	10 (32%)	
	Missing	2	0	2	0	
	***TYK2* rs280500**	157				0.37
	AA	106 (68%)	28 (74%)	61 (69%)	17 (55%)	
	AG	47 (30%)	10 (26%)	24 (27%)	13 (42%)	
	GG	4 (2.5%)	0 (0%)	3 (3.4%)	1 (3.2%)	
	***OAS1* rs1131454**	157				0.22
	GG	53 (34%)	13 (34%)	29 (33%)	11 (35%)	
	AG	70 (45%)	19 (50%)	42 (48%)	9 (29%)	
	AA	34 (22%)	6 (16%)	17 (19%)	11 (35%)	
	***TLR7* rs3853839**	157				0.80
	CC	112 (71%)	26 (68%)	61 (69%)	25 (81%)	
	CG	28 (18%)	8 (21%)	16 (18%)	4 (13%)	
	GG	17 (11%)	4 (11%)	11 (13%)	2 (6.5%)	
**Gene expression**	***TYK2***	157				0.80
	Mean (SD),<br>Median [IQR]	0.761 (0.377),<br>0.691 [0.522, 0.872]	0.701 (0.261),<br>0.665 [0.514, 0.847]	0.777 (0.406),<br>0.701 [0.514, 0.919]	0.788 (0.413),<br>0.649 [0.537, 0.858]	
	Range	0.176 - 2.547	0.319 - 1.566	0.176 - 2.547	0.398 - 2.155	
	TLR7	157				**0.001**
	Mean (SD),<br>Median [IQR]	0.417 (0.445),<br>0.308 [0.207, 0.497]	0.582 (0.719),<br>0.402 [0.313, 0.595]	0.390 (0.324),<br>0.294 [0.184, 0.498]	0.291 (0.185),<br>0.267 [0.158, 0.432]	
	Range	0.050 - 4.639	0.132 - 4.639	0.050 - 1.830	0.052 - 0.847	
	OAS1	157				**0.012**
	Mean (SD),<br>Median [IQR]	1.375 (1.206),<br>1.012 [0.668, 1.552]	1.146 (0.852),<br>0.902 [0.621, 1.409]	1.271 (1.152),<br>0.975 [0.654, 1.537]	1.952 (1.543),<br>1.357 [0.947, 2.749]	
	Range	0.078 - 6.575	0.168 - 3.575	0.078 - 6.491	0.136 - 6.575	

Extraction 2	Variable	N	Mild<br>N = 38[1]	Moderate<br>N = 88[1]	Severe<br>N = 31[1]	*p*-value[2]
**Housekeeping**	**Ct *HPRT***	157				0.15
	Mean (SD),<br>Median [IQR]	25.718 (0.638),<br>25.620 [25.273, 26.137]	25.575 (0.672),<br>25.408 [25.041, 26.094]	25.772 (0.617),<br>25.764 [25.364, 26.169]	25.741 (0.649),<br>25.527 [25.198, 26.155]	
	Range	24.301 - 27.591	24.611 - 27.255	24.301 - 27.591	24.986 - 27.419	
	**Ct *TBP***	157				**0.024**
	Mean (SD),<br>Median [IQR]	25.764 (0.651), 25.708<br>[25.295, 26.244]	25.519 (0.628), 25.424<br>[25.065, 25.929]	25.812 (0.624), 25.768<br>[25.370, 26.290]	25.928 (0.690), 25.990<br>[25.329, 26.303]	
	Range	24.201 - 27.603	24.201 - 26.948	24.435 - 27.055	24.806 - 27.603	
**Time**	**Days from symptom onset to extraction**	157				0.65
	Mean (SD),<br>Median [IQR]	13.688 (4.439), 13.000<br>[11.000, 16.000]	12.842 (4.779), 13.500<br>[8.000, 15.000]	13.659 (3.420), 13.000<br>[11.000, 16.000]	14.806 (6.177), 14.000<br>[11.000, 16.000]	
	Range	4.000 - 41.000	4.000 - 27.000	6.000 - 22.000	7.000 - 41.000	
**Treatments during hospitalization**	**Antihypertensives**	70 (45%)	14 (37%)	42 (48%)	14 (45%)	0.53
	ACEI	21 (13%)	5 (13%)	15 (17%)	1 (3.2%)	0.15
	ARB	20 (13%)	3 (7.9%)	14 (16%)	3 (9.7%)	0.44
	Diuretics	18 (11%)	4 (11%)	8 (9.1%)	6 (19%)	0.30
	Beta-blockers	25 (16%)	5 (13%)	17 (19%)	3 (9.7%)	0.42
	Calcium channel blockers	24 (15%)	3 (7.9%)	16 (18%)	5 (16%)	0.37
	Alpha blockers	2 (1.3%)	0 (0%)	2 (2.3%)	0 (0%)	>0.99
	**Anticoagulants**	152 (97%)	38 (100%)	85 (97%)	29 (94%)	0.30
	**Antiplatelets**	32 (20%)	7 (18%)	18 (20%)	7 (23%)	0.91
	**GCC**	145 (92%)	34 (89%)	82 (93%)	29 (94%)	0.72
	Dexamethasone	82 (52%)	27 (71%)	41 (47%)	14 (45%)	**0.028**
	Methylprednisolone	74 (47%)	7 (18%)	48 (55%)	19 (61%)	**<0.001**
	Other GCC	23 (15%)	8 (21%)	8 (9.1%)	7 (23%)	0.070
	**Immunosupressants**	54 (34%)	5 (13%)	31 (35%)	18 (58%)	**<0.001**
	Anakinra	1 (0.6%)	0 (0%)	1 (1.1%)	0 (0%)	>0.99
	Tocilizumab	35 (22%)	1 (2.6%)	20 (23%)	14 (45%)	**<0.001**
	Baricitinib	11 (7.0%)	0 (0%)	7 (8.0%)	4 (13%)	0.069
	Colchicine	9 (5.7%)	4 (11%)	5 (5.7%)	0 (0%)	0.21
	Lenalidomide	0 (0%)	0 (0%)	0 (0%)	0 (0%)	>0.99

Extraction 2	Variable	N	Mild<br>N = 38[1]	Moderate<br>N = 88[1]	Severe<br>N = 31[1]	*p*-value[2]
Treatments during hospitalization (cont)	**Antivirals**	12 (7.6%)	5 (13%)	6 (6.8%)	1 (3.2%)	0.29
	Remdesivir	8 (5.1%)	2 (5.3%)	5 (5.7%)	1 (3.2%)	>0.99
	Plitidepsin	1 (0.6%)	1 (2.6%)	0 (0%)	0 (0%)	0.44
	Entecavir	2 (1.3%)	1 (2.6%)	1 (1.1%)	0 (0%)	0.69
	Dovato	1 (0.6%)	1 (2.6%)	0 (0%)	0 (0%)	0.44
	Tenofovir	2 (1.3%)	0 (0%)	2 (2.3%)	0 (0%)	>0.99
	Aciclovir	2 (1.3%)	1 (2.6%)	1 (1.1%)	0 (0%)	0.69
	**Hyperimmune serum**	2 (1.3%)	1 (2.6%)	1 (1.1%)	0 (0%)	0.69
Laboratory parameters	**Lymphocytes (cells/µL) RR: 1000-4000**	157	38 (100%)	88 (100%)	31 (100%)	**<0.001**
	Mean (SD), Median [IQR]	1,708.854 (3,060.976), 1,370.000 [760.000, 2,040.000]	2,788.158 (5,981.350), 1,915.000 [1,170.000, 2,270.000]	1,510.795 (841.531), 1,460.000 [775.000, 2,080.000]	948.065 (595.804), 760.000 [520.000, 1,200.000]	
	Range	210.000 - 38,320.000	520.000 - 38,320.000	340.000 - 4,120.000	210.000 - 2,150.000	
	**Fibrinogen (mg/dL) RR: 150-400**	82 (52%)	17 (45%)	41 (47%)	24 (77%)	0.79
	Mean (SD), Median [IQR]	537.049 (164.167), 515.500 [431.000, 616.000]	499.176 (84.578), 514.000 [485.000, 546.000]	543.171 (148.080), 555.000 [431.000, 639.000]	553.417 (224.489), 479.000 [408.000, 671.000]	
	Range	213.000 - 1,071.000	274.000 - 618.000	235.000 - 818.000	213.000 - 1,071.000	
	Missing	75	21	47	7	
	**D-Dimer (µg/mL) RR: 0.15-0.5**	136 (87%)	32 (84%)	79 (90%)	25 (81%)	**<0.001**
	Mean (SD), Median [IQR]	2.770 (5.974), 0.995 [0.620, 1.685]	0.980 (1.201), 0.665 [0.435, 0.935]	2.293 (4.548), 1.060 [0.630, 1.630]	6.568 (10.566), 1.700 [0.950, 6.540]	
	Range	0.060 - 47.780	0.060 - 6.270	0.160 - 26.530	0.520 - 47.780	
	Missing	21	6	9	6	
	**LDH (U/L) RR: 135-225**	146 (93%)	35 (92%)	84 (95%)	27 (87%)	**<0.001**
	Mean (SD), Median [IQR]	301.998 (154.382), 265.500 [204.000, 348.000]	246.429 (101.369), 215.000 [188.000, 270.000]	277.331 (118.537), 250.500 [199.000, 324.000]	450.778 (213.348), 379.000 [317.000, 498.000]	
	Range	3.780 - 1,160.000	137.000 - 604.000	3.780 - 738.000	208.000 - 1,160.000	
	Missing	11	3	4	4	

Extraction 2	Variable	N	Mild<br>N = 38[1]	Moderate<br>N = 88[1]	Severe<br>N = 31[1]	*p*-value[2]
Laboratory parameters (cont)	**CRP (mg/dL)<br>RR: 0-0.5**	152 (97%)	35 (92%)	88 (100%)	29 (94%)	**<0.001**
	Mean (SD),<br>Median [IQR]	3.002 (4.342),<br>1.265 [0.475, 3.395]	1.279 (1.801),<br>0.900 [0.330, 1.450]	2.612 (3.071),<br>1.250 [0.495, 3.555]	6.265 (7.297),<br>2.540 [1.050, 8.080]	
	Range	0.070 - 28.300	0.090 - 10.290	0.110 - 12.910	0.070 - 28.300	
	Missing	5	3	0	2	
	**IL-6 (pg/mL)<br>RR: <6**	13 (8.3%)	1 (2.6%)	8 (9.1%)	4 (13%)	0.50
	Mean (SD),<br>Median [IQR]	140.092 (230.766),<br>16.200 [2.000, 149.000]	19.000 (NA),<br>19.000 [19.000, 19.000]	75.138 (143.962),<br>9.600 [0.950, 85.500]	300.275 (338.088),<br>299.550 [7.550, 593.000]	
	Range	0.000 - 600.000	19.000 - 19.000	0.000 - 409.000	2.000 - 600.000	
	Missing	144	37	80	27	
	**Ferritin (ng/mL)<br>RR: 30-400**	101 (64%)	23 (61%)	62 (70%)	16 (52%)	**<0.001**
	Mean (SD),<br>Median [IQR]	1,102.332 (1,082.115),<br>772.000<br>[359.000, 1,502.000]	757.239 (752.991),<br>395.000<br>[257.000, 1,301.000]	989.306 (917.138),<br>752.000<br>[317.000, 1,398.000]	2,036.375 (1,545.394),<br>1,643.000<br>[918.000, 2,324.000]	
	Range	0.500 - 5,681.000	0.500 - 2,985.000	60.000 - 4,954.000	364.000 - 5,681.000	
	Missing	56	15	26	15	
**Viremia**	**Viremia (copies/mL)**	126				**<0.001**
	Mean (SD),<br>Median [IQR]	1,809.130 (7,715.466),<br>110.000<br>[0.000, 340.000]	269.833 (1,028.624),<br>0.000<br>[0.000, 0.000]	846.502 (2,678.337),<br>110.000<br>[0.000, 230.000]	6,390.147 (16,133.178),<br>830.000<br>[150.000, 3,510.000]	
	Range	0.000 - 75,896.667	0.000 - 5,545.000	0.000 - 16,165.000	0.000 - 75,896.667	
	Missing	31	8	17	6	
**Demographic and clinical data**	**Sex**	157				0.36
	Male	82 (52%)	16 (42%)	49 (56%)	17 (55%)	
	Female	75 (48%)	22 (58%)	39 (44%)	14 (45%)	
	**Age (years)**	157				**0.045**
	<45	9 (5.7%)	5 (13%)	1 (1.1%)	3 (9.7%)	
	45-70	68 (43%)	17 (45%)	39 (44%)	12 (39%)	
	>70	80 (51%)	16 (42%)	48 (55%)	16 (52%)	
	**Hypertension**	84 (54%)	15 (39%)	52 (59%)	17 (55%)	0.13

Extraction 2	Variable	N	Mild<br>N = 38[1]	Moderate<br>N = 88[1]	Severe<br>N = 31[1]	*p*-value[2]
**Demographic and clinical data** (cont)	**Dementia**	6 (3.8%)	1 (2.6%)	4 (4.5%)	1 (3.2%)	>0.99
	**Obesity**	23 (15%)	3 (7.9%)	13 (15%)	7 (23%)	0.24
	**People living with HIV**	1 (0.6%)	1 (2.6%)	0 (0%)	0 (0%)	0.44
	**Diabetes mellitus**	31 (20%)	8 (21%)	20 (23%)	3 (9.7%)	0.28

Table S5. Significant variables associated with gene expression in the univariate analysis, adjusting by plate number and days from symptom onset to extraction date. ARB: angiotensin II receptor blockers; CRP: C-reactive protein; LDH: lactate dehydrogenase; AHT: antihypertensives; β: beta coefficient; CI: confidence interval. *: non-significant variables that were subsequently forced in the multivariate analyses.

	TLR7			***OAS1***			***TYK2***		
	**Variable**	**β (95% CI)**	***p*-value**	**Variable**	**β (95% CI)**	***p*-value**	**Variable**	**β (95% CI)**	***p*-value**
**Gene expression and SNPs**	*OAS1*	0.14 (0.04, 0.25)	**0.006**	*TYK2*	0.29 (0.20, 0.38)	**<0.001**	*OAS1*	0.22 (0.17, 0.27)	**<0.001**
	TYK2	-0.07 (-0.15, 0.02)	0.121*	*TLR7*	0.28 (0.17, 0.40)	**<0.001**			
				OAS1 rs1131454 (AG *vs* GG)	-0.20 (-0.51, 0.12)	0.219			
				OAS1 rs1131454 (AA *vs* GG)	-0.35 (-0.69, -0.02)	**0.04**			
				TLR7 rs3853839 (CG *vs* CC)	-0.06 (-0.37, 0.26)	0.714			
				TLR7 rs3853839 (GG *vs* CC)	-0.39 (-0.79, 0.01)	**0.057**			
				TYK2 rs280519 (AG vs GG)	-0.39 (-0.83, 0.05)	**0.084**			
				TYK2 rs280519 (AA vs GG)	0.01 (-0.46, 0.49)	0.951			
**COVID-19 severity and viral load**	Viremia	-0.12 (-0.23, -0.02)	**0.023**	Viremia	0.08 (-0.03, 0.20)	0.162*			
	Moderate *vs* mild	-0.45 (-0.65, -0.25)	**<0.001**						
	Severe *vs* mild	-0.69 (-0.99, -0.40)	**<0.001**						
**Demographic data and comorbidities**				Hypertension	0.27 (0.02, 0.52)	**0.035**	Age: 45-70 *vs* <45	0.17 (-0.11, 0.44)	0.23
							Age: >70 *vs* <45	0.28 (0.00, 0.56)	**0.047**
							Diabetes mellitus	0.21 (0.05, 0.37)	**0.011**
**Treatments prior to extractions**	ARB	0.48 (0.20, 0.77)	**<0.001**	ARB	0.39 (0.06, 0.71)	**0,019**	AHT	0.14 (0.03, 0.25)	**0.011**
	Glucocorticoids	-0.35 (-0.61, -0.10)	**0.006**	Glucocorticoids	-0.26 (-0.56, 0.05)	**0.102**	Diuretics	0.14 (-0.02, 0.29)	**0.088**
	Methylprednisolone	-0.27 (-0.45, -0.09)	**0.003**	Dexamethasone	0.19 (-0.00, 0.38)	**0.055**	Dexamethasone	0.10 (0.01, 0.20)	**0.034**

	TLR7			*OAS1*			*TYK2*		
	**Variable**	**β (95% CI)**	***p*-value**	**Variable**	**β (95% CI)**	***p*-value**	**Variable**	**β (95% CI)**	***p*-value**
**Treatments prior to extractions** (cont)	Calcium channel blockers	-0.42 (-0.70, -0.13)	**0.004**	Alpha-blockers	-1.24 (-2.30, -0.19)	**0.02**			
	Immunosuppressants	-0.24 (-0.45, -0.04)	**0.021**						
	Tocilizumab	-0.33 (-0.57, -0.09)	**0.006**						
	Lenalidomide	1.79 (0.16, 3.43)	**0.031**						
**Laboratory findings**	Fibrinogen	-0.19 (-0.32, -0.07)	**0.003**				Fibrinogen	-0.06 (-0.12, 0.01)	**0.082**
	Lymphocytes	0.22 (0.12, 0.32)	**<0.001**						
	D-dimer	-0.11 (-0.20, -0.02)	**0.019**						
	CRP	-0.15 (-0.25, -0.06)	**0.002**						
	LDH	-0.30 (-0.40, -0.21)	**<0.001**						
	Ferritin	-0.17 (-0.29, -0.05)	**0.006**						
